# Modulating Strategies of the Intestinal Microbiota in Colorectal Cancer

**DOI:** 10.3390/nu17223565

**Published:** 2025-11-14

**Authors:** María José García Mansilla, María Jesús Rodríguez Sojo, Andreea Roxana Lista, Ciskey Vanessa Ayala Mosqueda, Jorge García García, Julio Gálvez Peralta, Alba Rodríguez Nogales, Antonio Jesús Ruiz Malagón, María José Rodríguez Sánchez

**Affiliations:** 1 Department of Pharmacology, Centro de Investigación Biomédica (CIBM), University of Granada, 18071 Granada, Spain; garciamansillamariajose@gmail.com (M.J.G.M.); mariajesus.rodriguez.sojo@gmail.com (M.J.R.S.); jgalvez@ugr.es (J.G.P.); ajruiz@ugr.es (A.J.R.M.); mjrs2188@gmail.com (M.J.R.S.); 2 Instituto de Investigación Biosanitaria de Granada (ibs.GRANADA), 18012 Granada, Spain; andreea99.lista@gmail.com (A.R.L.); ciskey@correo.ugr.es (C.V.A.M.); 3 CIBER de Enfermedades Hepáticas y Digestivas (CIBER-EHD), Instituto de Salud Carlos III, 28029 Madrid, Spain

**Keywords:** microbiota, colorectal cancer, therapeutic approaches, probiotics, prebiotics, postbiotics

## Abstract

**Background/Objectives**: Colorectal cancer (CRC) accounts for nearly 10% of global cancer cases and is the second leading cause of cancer-related mortality. While age and genetics are non-modifiable risk factors, nutrition and its impact on gut microbiota are emerging as key determinants in CRC prevention and management. We aimed to systematically evaluate recent evidence on the role of diet and microbiota-targeted interventions—including probiotics, prebiotics, synbiotics, and postbiotics—in modulating CRC risk and therapeutic outcomes. **Methods**: A structured literature search was performed in PubMed, ResearchGate, Scopus, and ScienceDirect up to July of 2025. Reference lists of relevant reviews and clinical trials were also screened. A total of 36 studies were selected according to PRISMA guidelines. Data were extracted on dietary exposures, microbiota modulation, metabolite profiles, and CRC-related outcomes. Evidence quality was assessed using appropriate appraisal tools for observational and interventional designs. **Results**: Western-type diets were consistently associated with microbiota dysbiosis, the enrichment of pro-inflammatory and genotoxic taxa, and elevated CRC risk. Diets rich in fiber and polyphenols enhanced commensals producing short-chain fatty acids (e.g., butyrate), with anti-inflammatory and antineoplastic effects. Probiotics, prebiotics, and postbiotics demonstrated potential to restore microbial balance, improve epithelial integrity, and enhance tolerance to conventional therapies. **Conclusions**: Current evidence supports a complex interplay between nutrition, the gut microbiota, and CRC, with strong translational potential. Microbiota-modulating nutritional strategies, particularly fiber-rich diets and synbiotics, show the most consistent microbiota-related benefits in CRC prevention and represent promising adjuncts to standard therapies. However, much of the available research is still based on preclinical models. Therefore, there is a pressing need for well-designed clinical studies in human populations to validate these findings and inform evidence-based guidelines.

## 1. Introduction

CRC represents ~10% of all cancer cases and is the second leading cause of cancer-related death worldwide [1]. Risk is driven by age, genetic predisposition, and environmental factors. Incidence rises sharply after 50 years and is strongly influenced by lifestyle—unhealthy diets, alcohol, tobacco, and low physical activity [2]. Familial clustering is observed in ~30% of cases, yet only 5–16% are attributable to germline variants in CRC-predisposing genes, including APC, KRAS, BRAF, TGFBR2, MUTYH, STK11, and TP53 [3,4,5].

The gut microbiota has increasingly been recognized as a central determinant in the pathogenesis and progression of CRC. Emerging evidence indicates that microbial dysbiosis can compromise the intestinal barrier, modulate immune surveillance, and produce metabolites that drive oncogenic signaling in the colonic epithelium. Specifically, this dysbiosis is characterized by reduced diversity and enrichment of pro-carcinogenic taxa such as *Fusobacterium nucleatum*, *Escherichia coli*, *Parvimonas micra*, and *Bacteroides fragilis* [6,7,8].

As research advances, the microbiota has shown to be not only implicated in causation but also in therapeutic responsiveness, with studies exploring how modulation of gut microbes may enhance checkpoint inhibitor efficacy in CRC [9,10]. Recent studies have proved that targeting specific microbial communities can attenuate inflammation, enhance anti-tumor immunity, and improve responses to immunotherapy, particularly immune checkpoint inhibitors [11,12]. Moreover, microbial metabolites such as short-chain fatty acids (SCFAs), especially butyrate, have been linked to anti-inflammatory and anti-proliferative effects in the colonic epithelium [13,14].

In this context, nutritional interventions—including dietary compounds, probiotics, prebiotics, postbiotics, and synbiotics—have shown promising potential not only in restoring microbial balance and reducing inflammation but also in complementing conventional therapies [15,16,17,18], which remain limited by recurrence, toxicity, post-surgical complications, and therapeutic resistance [19,20,21].

Given the growing global burden of CRC, diet–microbiota interactions play a central role in disease risk, development, and therapeutic response. Alongside the increasing availability of nutritional and microbiota-targeted interventions, a systematic synthesis of the current evidence is urgently needed. While previous reviews have examined the relationship between the gut microbiota and CRC, as well as different strategies for its management, many have primarily focused on the use of specific probiotics, prebiotics or diet interventions in preclinical models. In contrast, the present review offers a broader and more integrative perspective by encompassing a wider spectrum of microbiota-targeted strategies—including probiotics, prebiotics, synbiotics, postbiotics, and dietary interventions. Notably, this review goes beyond experimental data to incorporate current clinical evidence and ongoing trials, thereby providing an updated and translational overview of how these interventions may modulate CRC risk and influence therapeutic outcomes.

## 2. Materials and Methods

This systematic review was conducted to evaluate the efficacy of different microbiota-modulating strategies with favorable outcomes in the context of CRC. The study focused on interventions such as dietary modifications, and the use of probiotics, prebiotics, postbiotics, as well as other indirect methods.

### 2.1. Search Strategy

The methodology of this review followed the Preferred Reporting Items for Systematic Reviews and Meta-Analyses (PRISMA) guidelines, implementing a rigorous process that encompassed database searches, study screening, eligibility evaluation, and final selection of relevant articles to ensure transparency and methodological rigor in scientific research.

A comprehensive bibliographic search was performed in PubMed, ResearchGate, Scopus, and ScienceDirect to identify relevant literature published until July of 2025. Boolean operators were used to optimize the search, combining key terms such as “gut microbiota”, “dysbiosis”, “colorectal cancer”, “probiotics”, “prebiotics”, “postbiotics”, and “dietary interventions”. Additional filters were applied to exclude duplicate records across databases, non-relevant studies, and articles not published in open access format.

### 2.2. Study Selection Criteria

**Figure 1:**
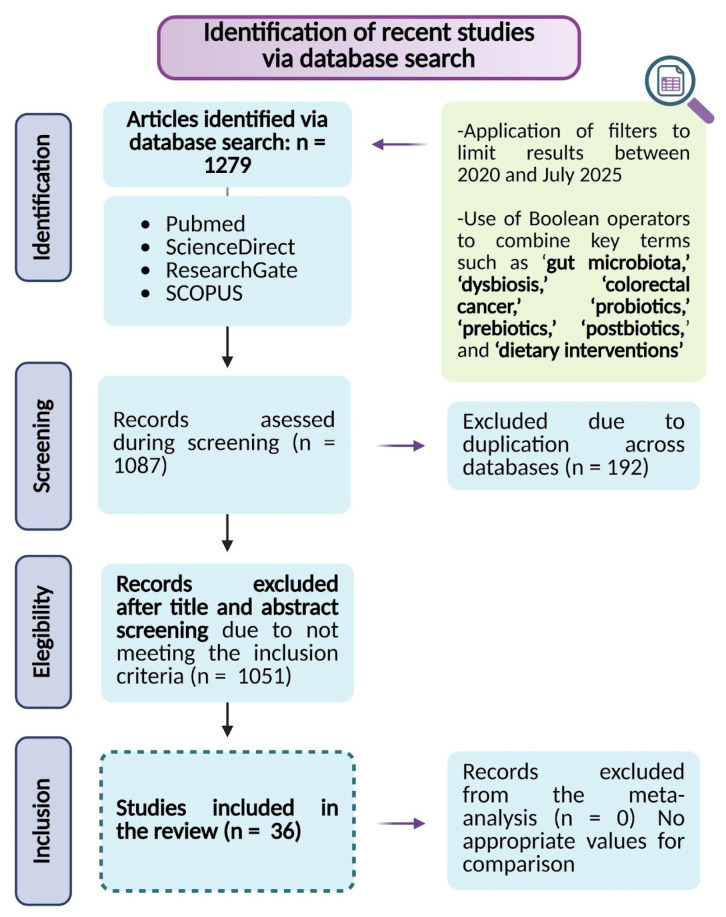
Flowchart of study selection following the PRISMA guidelines.

The search was restricted to peer-reviewed articles published in English within the last five years, ensuring the inclusion of high-quality, up-to-date evidence. The eligibility criteria were designed to identify experimental and clinical studies evaluating microbiota-targeted interventions in CRC (Figure 1).

Eligible studies included preclinical research using cellular or animal models of CRC, clinical trials, and randomized controlled trials assessing the effects of gut microbiota modulation on CRC progression and treatment response. Additionally, studies that reported defined outcome measures related to microbiota modulation, CRC prognosis, immune response, or therapy effectiveness were considered.

To ensure a more precise scope for the review, exclusion criteria were systematically applied. Articles were excluded if they consisted of opinion pieces, theoretical models, or reviews lacking original experimental data. Additionally, studies without a clearly defined methodology or well-established outcome measures related to gut microbiota modulation in CRC were excluded. Furthermore, studies that solely examined gut microbiota changes without assessing their impact on CRC pathophysiology or treatment outcomes were also omitted.

A screening process was then conducted to remove irrelevant studies and duplicate records across databases, ensuring the selection of the most relevant literature for this review.

### 2.3. Quality Assessment, Data Extraction and Grouping

Study selection and data extraction were conducted independently by two reviewers. Any discrepancies were resolved through discussion, and if consensus could not be reached, a third reviewer was consulted to make the final decision. Inter-rater reliability was not assessed quantitatively, but all disagreements were documented and resolved prior to inclusion in the analysis. For the data extraction process, a checklist was developed based on the predefined selection criteria. This checklist captured information such as the year of publication, study authors, study type—clinical or preclinical—, and sample size, among other variables.

To evaluate potential bias, the Cochrane Risk of Bias tool was applied for randomized controlled trials [22], while the SYRCLE Risk of Bias tool was used for animal studies [23]. Additionally, the overall quality of evidence was appraised in accordance with the GRADE methodology [22].

This systematic review was prospectively registered with PROSPERO (Registration ID: 1141584), ensuring adherence to predefined methods and minimizing reporting bias.

## 3. Results

The results of this systematic review underscore the translational relevance of microbiota-directed strategies as innovative approaches to improve clinical outcomes and guide the development of future preventive and therapeutic interventions in CRC management. An initial comprehensive database search identified 1279 records based on predefined inclusion and exclusion criteria. Following the screening of titles and abstracts, 1051 records were excluded, and 192 duplicate entries were removed, resulting in 36 studies considered for inclusion in the final analysis. The study selection process is detailed in Figure 1. This selection encompasses evidence from in vitro studies, CRC animal models, and human clinical trials. Collectively, this integrated body of research highlights that modulation of the gut microbiota can exert a significant impact on CRC progression and patient response to treatment, thereby providing robust support for microbiota-targeted strategies as promising interventions to mitigate gut dysbiosis and enhance the efficacy of existing CRC therapies.

### 3.1. Translational Insights into Prebiotic Effects on Gut Microbiota and Colorectal Carcinogenesis: Experimental and Clinical Evidence

Prebiotics are non-digestible food components—typically fibers or complex carbohydrates—that selectively stimulate the growth and/or metabolic activity of beneficial bacterial taxa such as *Faecalibacterium*, *Roseburia*, *Ruminococcus*, and *Akkermansia* [24]. In the context of CRC, prebiotics have attracted considerable scientific interest due to their capacity to modulate gut microbiota composition and functionality, thereby influencing key immunological, inflammatory, and metabolic processes implicated in CRC initiation and progression.

In vitro studies have provided foundational evidence of the antitumor effects of various prebiotics. For instance, propyl propane thiosulfonate (PTSO), a bioactive compound derived from *Allium* species such as garlic and onion, demonstrated selective and dose-dependent cytotoxicity in CRC cell lines —HT29 and T84—, reducing cell viability by up to 44% when delivered in a microencapsulated form. Beyond its direct cytotoxic effects, PTSO was shown to beneficially modulate gut microbiota composition by increasing microbial diversity, enhancing the production of SCFAs, and attenuating oxidative stress—all of which may contribute to CRC risk reduction [25]. Similarly, ginsenosides—the principal active compounds in ginseng—such as 20R-Rg3 and Rg5-BG, have been reported to induce apoptosis in CRC cells through activation of the caspase-3/Bax/Bcl-2 signaling pathway, an effect closely associated with microbiota modulation [26].

In vivo preclinical studies have further supported the therapeutic potential of prebiotics. Rice bran, a dietary source rich in phytochemicals and prebiotic fibers, promoted the proliferation of beneficial bacteria—including *Alloprevotella*, *Prevotellaceae*, *Ruminococcus*, *Roseburia*, *Butyricicoccus*, *Flavonifractor*, and *Oscillibacter*—while simultaneously reducing potentially pathogenic genera such as *Clostridium*, *Escherichia–Shigella*, and *Citrobacter* in CRC murine models [27,28]. Additionally, a CRC humanized gnotobiotic mice model transplanted with fecal microbiota from CRC survivor patients consuming rice bran led to a significant reduction in neoplastic lesions and favorable modulation of colonic metabolite profiles, including decreased levels of carcinogenesis-associated metabolites such as trimethylamine N-oxide and tartrate. These changes were accompanied by reduced concentrations of oxidized glutathione, trigonelline, quinolinate, 4-hydroxyphenylpyruvate, and proline, and were linked to the regulation of key metabolic pathways involving methionine, glutathione, secondary bile acids, and phenolic compounds, ultimately supporting an antioxidant and anti-inflammatory effect [28].

Further, the ginsenoside Rh4, a type of saponin found in ginseng—*Panax ginseng* and *Panax notoginseng*—has demonstrated significant inhibitory effects on CRC development in mice by promoting the growth of *Akkermansia muciniphila*, a commensal bacterium known to modulate the TLR4–NF-κB inflammatory signaling pathway. Rh4 administration was associated with enhanced production of ursodeoxycholic acid via the activation of 7α-hydroxysteroid dehydrogenase, which subsequently activated FXR signaling and contributed to CRC suppression. Additionally, Rh4 increased gut microbiota diversity, restored intestinal barrier integrity, and downregulated the mRNA expression of pro-inflammatory cytokines IL-6 and IL-1β, as well as the inflammatory mediator COX-2 [29].

Clinical studies have also provided valuable insights into the translational relevance of prebiotics in CRC. For example, supplementation with rice bran in individuals at elevated CRC risk was associated with an increased abundance of beneficial bacterial genera, including *Firmicutes*, *Lactobacillus*, *Bifidobacterium*, *Prevotella*, and *Lactobacillales*, supporting its prebiotic potential in human populations [30]. Additionally, a study in CRC patients receiving radiotherapy evaluated the administration of the prebiotic oat bran alone and its combined use with the probiotic *Lactobacillus plantarum* HEAL19 and blueberry husks, showing notable benefits in both separate and combined interventions. Participants receiving the prebiotic intervention exhibited reduced white blood cell counts compared to controls, while the synbiotic group showed additional reductions in inflammation and fibrosis. Notably, despite the overall reduction in bacterial diversity typically induced by radiotherapy, the synbiotic group maintained a higher proportion of mucosa-associated bacterial species, highlighting the enhanced protective effect of the combined intervention on gut microbiota composition [31].

Moreover, recent clinical research has explored the use of prebiotics as an adjunct to conventional oncological treatments. An ongoing clinical trial is evaluating whether supplementation with xylooligosaccharides—non-digestible carbohydrates that serve as a substrate for *Bifidobacteria* and *Lactobacilli*—can improve the efficacy of chemotherapy, reduce treatment-related adverse effects, and enhance patient quality of life in CRC [32].

All the results mentioned above involving the use of prebiotics are summarized in the following Table 1.

### 3.2. Probiotic-Driven Modulation of Gut Microbiota in CRC: Integrative Experimental and Clinical Perspectives

The modulatory potential of probiotics on the gut microbiota and their therapeutic implications in CRC has been extensively explored across several models and/or studies. In vitro studies utilizing human CRC cell lines, such as Caco-2 and HIEC-6, have demonstrated that specific strains of *Lacticaseibacillus*, including *Lacticaseibacillus rhamnosus* SD11 and *Lacticaseibacillus paracasei* SD1, effectively provide beneficial anti-carcinogenesis effects through the production of SCFAs, including inhibition of pathogen-related CRC, the stimulation of antimicrobial peptides or the inhibition of cancer cells [33]. Moreover, these strains have also been shown to enhance the expression of antimicrobial peptides like human β-defensin (hBD) and anti-inflammatory cytokines such as IL-10, suggesting a dual role in both direct tumor suppression and immune modulation [34]. Supporting these findings, *Lactobacillus gallinarum* has exhibited similar selective antitumor activity in vitro and in patient-derived organoids, indicating a robust effect across different CRC models [35].

Complementary evidence from in vivo animal models further substantiates the capacity of probiotics to modulate gut microbial ecology and suppress colorectal tumor development. Administration of *Lactobacillus gallinarum* in murine CRC models resulted in decreased abundance of pro-tumorigenic bacterial taxa including *Alistipes*, *Dorea*, and *Parabacteroides*, coupled with increased colonization by beneficial commensals such as *Lactobacillus helveticus* and *Lactobacillus reuteri* [35]. Similarly, multi-strain probiotic formulations containing *Bifidobacterium*, *Lactobacillus*, and *Enterococcus* enhanced microbial richness and diversity in xenograft models, favoring the proliferation of *Bacteroidota* and *Proteobacteria* while reducing taxa associated with CRC progression, such as *Ruminococcus* and *Verrucomicrobia* [36]. Furthermore, in a murine model of CRC (ApcMin/+), the synergistic combination of probiotics (*Bifidobacterium bifidum* and *Lactobacillus gasseri*) with the bioactive dietary compound quercetin significantly reduced tumor development compared to a standard diet and to a diet supplemented with probiotics alone. This effect was associated with the downregulation of the canonical Wnt/β-catenin signaling pathway, a key driver of CRC tumorigenesis [37].

Further animal studies highlight the context-dependent efficacy of probiotic interventions. Niechciał et al. evaluated two probiotic compositions in murine CRC models, demonstrating that a combination of *Lactobacilli* and *Bifidobacteria*—Composition I—significantly reduced tumor burden in an orthotopic MC-38 model by stabilizing the gut microbiota and promoting butyrate-producing taxa such as *Lachnospiraceae* NK4A214. In contrast, a formulation containing solely *Bifidobacteria*—Composition II—was more effective in the inflammation-driven AOM/DSS model, markedly suppressing colonic inflammation and preventing tumor formation altogether [38]. These findings underscore the necessity of tailoring probiotic strategies to specific pathological contexts to maximize therapeutic outcomes.

Additional targeted probiotic interventions have demonstrated promising antitumor and immunomodulatory effects in preclinical studies. For instance, *Parabacteroides johnsonii* administration in AOM/DSS-induced CRC mice suppressed tumor growth, improved histopathological parameters, and restored gut microbial diversity, notably increasing beneficial species such as *Bifidobacterium pseudolongum* and *Lactobacillus*. This intervention also modulated critical metabolic pathways including amino sugar metabolism and tryptophan biosynthesis, which are relevant to tumor microenvironment regulation [39]. The emerging probiotic candidate *Akkermansia muciniphila* has shown similar efficacy in attenuating intestinal inflammation via NF-κB pathway modulation, promoting apoptosis, and shifting microbial communities to favor taxa such as *Muribaculaceae*, while reducing pro-inflammatory *Bacteroides* and *Parasutterella* species [40]. In addition, the butyrate-producing bacterium *Clostridium butyricum* has demonstrated antitumour capacity by inducing apoptosis and inhibiting Wnt/β-catenin signaling, effects accompanied by beneficial modulation of the composition of the gut microbiota, in studies that combined murine models of intestinal cancer and in vitro studies with human CRC cell lines [41]. These effects were mirrored by administration of probiotic fermented milk containing *Bifidobacterium animalis* ssp. *lactis* BX-245, which enhanced *Akkermansia* levels within the tumor microenvironment, strengthened the intestinal barrier, and elevated systemic immune mediators such as IL-2 and IFN-γ [42].

The translational relevance of these findings is supported by clinical studies demonstrating probiotic efficacy in CRC patients. Supplementation with *Lactobacillus paracasei* SD1 and *Lacticaseibacillus rhamnosus* SD11 increased the abundance of SCFA-producing bacteria and elevated fecal concentrations of butyrate, acetate, and propionate, while concurrently reducing pathogenic bacteria such as *Fusobacterium nucleatum* and *Porphyromonas gingivalis*. These microbiota modifications were associated with increased levels of anti-inflammatory cytokines IL-10 and IL-12, indicating a beneficial immunomodulatory effect [43].

From a therapeutic standpoint, the interaction between microbiota and conventional CRC treatments deserves special consideration. Certain microbial species can directly alter drug metabolism, as seen with *Clostridium perfringens* reducing trifluridine efficacy, an effect counteracted by uridine supplementation [44]. Such findings highlight the dual role of the microbiota as both a therapeutic target and a modulator of pharmacological responses, underscoring the need for integrative nutritional strategies in CRC care.

Additionally, probiotic interventions have shown promise in mitigating chemotherapy-associated dysbiosis. In murine models receiving 5-fluorouracil, administration of resistant starch-encapsulated probiotics reduced microbial imbalance, enhanced bacterial abundance, and elicited anti-inflammatory and pro-apoptotic responses [45]. Parallel clinical data from a randomized study involving 100 CRC-patients demonstrated that a probiotic cocktail containing *Bifidobacterium infantis*, *Lactobacillus acidophilus*, *Enterococcus faecalis*, and *Bacillus cereus* restored microbial diversity disrupted by chemotherapy and increased SCFA-producing taxa. This intervention also alleviated gastrointestinal symptoms commonly observed during treatment, including diarrhea and abdominal distension [46].

Taken together, these convergent lines of evidence from in vitro, in vivo, and clinical studies underscore the critical role of probiotics in modulating gut microbiota composition and function, exerting antitumor effects, and enhancing host immune responses in CRC (Table 2). The context-dependent efficacy observed across different models highlights the importance of personalized probiotic strategies tailored to disease stage and pathophysiology. These findings pave the way for integrating probiotics as adjunctive therapeutic agents to improve CRC outcomes through microbiota-targeted interventions.

### 3.3. Postbiotic-Driven Modulation of Gut Microbiota and Its Therapeutic Implications in CRC: From Bench to Bedside

In addition to probiotics and prebiotics, evidence gathered in this systematic review indicates that metabolites produced by gut microbiota—postbiotics—play a significant role in regulating dysbiosis and supporting a healthy microbial balance. These bioactive compounds have demonstrated antioxidant, anti-inflammatory, and antitumor properties, and are emerging as promising adjunctive strategies for CRC and other diseases.

In vitro studies have highlighted the therapeutic potential of metabolites derived from *Lactobacillus gallinarum*, which exhibited pronounced pro-apoptotic effects against CRC cell lines and patient-derived organoids. These cytotoxic effects appear to be mediated through the biosynthesis of L-tryptophan and its subsequent conversion to indole-3-lactic acid, a compound with documented antitumor activity [35]. Additionally, the antitumor efficacy of SCFAs—key postbiotic molecules—has been demonstrated in assays with human CRC cell lines—HT29, T84, and HCT116—where butyrate showed notable cytotoxicity in HCT116 cells.

Complementary in vivo evidence further substantiates the role of postbiotics in modulating gut microbiota and suppressing colorectal tumorigenesis. In a murine CRC model, administration of *Lactobacillus gallinarum* metabolites resulted in significant reductions in tumor incidence and size. These effects were accompanied by beneficial alterations in gut microbiota composition, including increased abundance of *Lactobacillus helveticus* and *Lactobacillus reuteri*, and decreased levels of pro-inflammatory or pro-tumorigenic taxa such as *Alistipes*, *Parabacteroides*, and *Ruminococcus* [35]. Similarly, polyhydroxybutyrate (PHB), a polyhydroxyalkanoate produced by various microorganisms, demonstrated a favorable safety and efficacy profile in a rat CRC model. PHB supplementation promoted the proliferation of beneficial *Bacillota*—previously named *Firmicutes* families, including *Lactobacillaceae*, *Peptococcaceae*, and *Eubacteriaceae*—and increased SCFA production, notably of butyrate and 3-hydroxybutyrate. These microbiota shifts were associated with a 58% reduction in tumor area and a 34% decrease in tumor number, alongside the establishment of an anti-inflammatory intestinal milieu and enhanced overall microbial diversity. Importantly, PHB treatment also led to a notable reduction in *Proteobacteria*, a phylum often linked to intestinal inflammation [47].

Clinical data reinforce the translational relevance of these findings. In a randomized controlled trial involving individuals in stage II or III of CRC, administration of metabolites produced by *Lacticaseibacillus paracasei* SD1 and *Lacticaseibacillus rhamnosus* SD11 promoted a shift in gut microbiota composition toward a healthier profile, closely resembling the effects previously attributed to the live probiotic strains themselves. This intervention resulted in elevated fecal concentrations of SCFAs—butyrate, acetate, and propionate— an increased abundance of SCFA-producing bacteria, and higher relative proportions of *Bacillota*, *Bacteroides*, and *Prevotella*. Concurrently, levels of anti-inflammatory cytokines IL-10 and IL-12 were significantly increased, while pro-inflammatory cytokines and pathogenic CRC-associated taxa such as *Fusobacterium* were reduced [43].

Taken together, these converging lines of evidence highlight the potential of postbiotics to modulate gut microbiota composition, attenuate inflammation, and exert antitumor effects in CRC (Table 3). The consistent outcomes observed across in vitro assays, animal models, and clinical trials support their future integration as complementary therapeutic agents in CRC management.

### 3.4. Feeding the Microbiome: Dietary Influence on Microbial Composition

In addition to the previously mentioned strategies, there are other approaches with potential to modulate the gut microbiota and, consequently, influence CRC outcome (Table 4). Among them, dietary modification stands out as particularly relevant, existing different studies that inversely related the effects of maintaining a healthy diet with CRC risk.

Existing studies suggest a link between CRC and the presence of *Escherichia coli* carrying the polyketide synthase gene (*Escherichia coli* pks+) in the gut. This gene encodes enzymes involved in the biosynthesis of polyketides, a diverse group of natural products including colibactin, a genotoxic secondary metabolite that alkylates the host cell DNA, which leads to genomic instability, being potentially involved in the development of CRC. In this regard, studies have evaluated fecal concentrations of *Escherichia coli* pks+ in relation to dietary patterns, showing that certain components such as green tea, eggs, and manganese may reduce its presence [48].

On the other hand, research based on fecal sample analysis of CRC survivors has shown how the consumption of fruits and vegetables increases microbial diversity, raises the abundance of *Firmicutes*, and decreases levels of *Bacteroidota* and *Fusobacterium nucleatum*. These effects are associated with the upregulation of the anabolic pathway for the biosynthesis of dTDP-N-acetylviosamine, as well as increased levels of certain SCFAs, amino acids, nucleic acids, and the activation of catabolic pathways involved in sugar degradation [49].

As previously described, it is well known that maintaining a healthy lifestyle, including a varied and balanced diet, can promote the maintenance of an optimal gut microbiota variety and functionality. In this context, recent studies have focused on examining the effects of specific dietary compounds that support microbiota modulation in various diseases, and specifically in CRC. Below, we detail the most relevant compounds that have been examined up to date.

A well-studied dietary compound is curcumin; a polyphenol derived from the *Curcuma longa* plant. In this line, recent research demonstrated that oral administration of curcumin was able to suppress tumor progression and increased infiltration of CD8+ T cells into tumor tissues. Specifically, this compound has shown to modulate the gut microbiota of CRC mice, partially restoring microbial diversity and composition, along with altering serum metabolite profiles. Notably, curcumin elevated levels of SCFA-producing bacteria such as *Lactobacillus* and *Kineothrix*. Along the study, a fecal microbiota transplantation (FMT) experiment showed how mice receiving fecal material from curcumin-treated donors had marked improvement in CRC symptoms, including reduced tumor growth, enhanced CD8+ T cell infiltration into tumors, and increased ferroptosis. Remarkably, when gut microbiota was depleted via antibiotic treatment, the antitumor efficacy of curcumin was abolished, suggesting that the therapeutic effects were dependent on the presence of gut microbiota [50].

Another study carried out by Deng al cols. [51] showed how curcumin administration led to the recovery of colon length and architecture, along with a marked reduction in tumor development in mice with AOM/DSS-induced CRC. This study revealed that both the diversity and abundance of core and total gut microbiota were significantly diminished in CRC mice compared to the control group. Specifically, curcumin showed to suppress the overgrowth of potentially harmful genera such as *Ileibacterium*, *Monoglobus*, and *Desulfovibrio*, which were elevated in the CRC model. Conversely, the levels of beneficial bacteria—including Clostridia_UCG-014, *Bifidobacterium*, and *Lactobacillus*—which were reduced in CRC mice, were significantly increased after curcumin intervention.

Following with curcumin interventions, a recent study testing a combination of curcumin with turmeric essential oil and a tocotrienol-rich fraction of vitamin E reported a significantly reduced proliferation of CRC cells—HCT-116 and HT-29—in vitro and suppressed the growth of HCT-116-derived xenografts in vivo. This combination led to a significant increase in *Lactobacillaceae* and *Bifidobacteriaceae*, as well as *Clostridium* cluster XIVa, contributing to an anti-inflammatory environment. Additionally, the relative abundance of the phyla *Bacteroidota* and *Firmicutes* was reduced [52].

We now turn our attention to another compound that is extensively used and thoroughly studied, ginger—*Zingiber officinale*—. This dietary compound has been widely used for centuries as a natural remedy for various gastrointestinal problems. Its active compounds, such as gingerol, zingerone or shogaol among others, have shown to have powerful anti-inflammatory and antioxidant properties. Moreover, in recent years scientific studies have explored its potential role in preventing and treating CRC. Current literature suggests that ginger may help to slow the growth of cancer cells in the colon and reduce inflammation in the digestive tract [53,54,55]. Interestingly, recent preclinical models have suggested that changes in the gut microbiome could be mediating these beneficial effects. In this way, an in vitro study showed how digested ginger extract was able to alter the structure of the fecal microbiota, enhancing the growth of beneficial bacterial populations such as *Bifidobacterium* and *Enterococcus* as well as to increase levels of SCFAs and a corresponding reduction in pH [56]. Another study aimed to assess how fresh ginger-derived juice affects gut microbiota composition and function. Specifically, a crossover intervention with 123 participants consuming fresh ginger juice showed an increased microbial species richness and altered key bacterial ratios, including a decreased *Prevotella*-*Bacteroides* ratio and reductions in pro-inflammatory *Ruminococcus*. Furthermore, trends toward increased *Firmicutes*-*Bacteroidota* ratio, *Proteobacteria*, and anti-inflammatory *Faecalibacterium* were also observed [57].

**Table 4:** Evidence of the effect of different dietary components on the modulation of the gut microbiota and on parameters associated with CRC [36,48,49,50,51,52,53,54,56,57,58,59,60,61,62].

Outcome	Dietary Compound	Model of CRC Used
↓ *E. coli* pks+ in fecal samples	Green tea and manganese intake	Healthy individuals (human)
↑ Microbial diversity	Curcumin Curcumin + Vitamin E tocotrienols Berberine	Murine
	Fruit and vegetable	CRC survivors (human)
– Microbial diversity	Ginger derivatives	Individuals previously diagnosed with colorectal adenoma
↑ Beneficial bacteria	Curcumin P127-MLL@Gins nanoparticle Berberine HPS	Murine
	Ginger derivatives	In vitro simulated digestive and fermentative processes of ginger
	Ginger derivatives	Healthy individuals (human)
**↓** Pathogenic species	Curcumin P127-MLL@Gins nanoparticle Berberine HPS	Murine
	Ginger derivatives	Individuals previously diagnosed with colorectal adenoma
↑ SCFAs	Fruit and vegetable	CRC survivors (human)
	Ginger derivatives	In vitro simulated digestive and fermentative processes of ginger
↓ Cell viability/proliferation	Curcumin Curcumin + Vitamin E tocotrienols Ginger derivatives Berberine	Cell lines
	Curcumin P127-MLL@Gins nanoparticle HPS	Murine
↑ Immune response	Curcumin P127-MLL@Gins nanoparticle	
**↓** Inflammation	Curcumin + Vitamin E tocotrienols Berberine HPS	
	Ginger derivatives	Healthy individuals (human)
**↓** Tumor growth	Curcumin Curcumin + Vitamin E tocotrienols Berberine HPS	Murine
**↓** Number/size of tumors	P127-MLL@Gins nanoparticle	
↑ Colon length	Curcumin HPS	
**↓** Tissue damage	Berberine HPS	
↑ Body weight	HPS	
**↓** pH	Ginger derivatives	In vitro simulated digestive and fermentative processes of ginger

Focusing specifically on cancer, P127-MLL@Gins, an oral nanoparticle composed of magnetic mesoporous silica loaded with 6-gingerol, coated with Pluronic F127 and mulberry leaf-derived lipids, has shown to promote intestinal release, and selectively target CRC tumor cells [58]. Upon exposure to alternating magnetic fields, it exhibited potent pro-apoptotic activity mediated by high mobility group box 1, immunogenic cell death, increased reactive oxygen species, and suppressed proliferation. It also promoted M1 macrophage polarization, CD4+ and CD8+ T cell activation, Treg reduction, and elevated TNF-α and IFN-γ levels, enhancing the immune response. These effects resulted in an in vivo reduction in tumor number and size up to 54.5-fold compared to the control group. Additionally, microbial diversity and richness increased, along with a higher *Firmicutes/Bacteroidota* ratio, greater abundance of *Bacillus*, and a decrease in harmful genera such as *Alloprevotella* and *Bacteroides*. At the metabolic level, key pathways related to glutamate, essential fatty acids, sphingolipids, tyrosine, and tryptophan were activated.

At clinical level, 68 participants were randomized in a study to receive either ginger or placebo daily for 6 weeks. After ginger supplementation were observed significant reductions in the relative abundances of the genera *Akkermansia*, *Bacteroides*, and *Ruminococcus* [59].

In addition to the mentioned above, there are other dietary compounds capable of modulating the gut microbiota. Among them, berberine—a bioactive compound found in *Rhizoma Coptidis*—stands out for its antitumor properties and beneficial effects against CRC development. The reviewed studies indicate that berberine treatment, both in vitro and in vivo in CRC models, promotes epithelial damage repair, limits metastasis, and regulates various metabolic pathways and inflammation, while concurrently enhancing microbiota composition and promoting SCFA production [36,60,61]. Therefore, the mechanism of action of berberine is associated with the reversal of intestinal dysbiosis, promoting the growth of beneficial species such as *Akkermansia* and reducing the presence of pathogenic species such as *Escherichia*.

In preclinical animal models of CRC, berberine has shown beneficial effects on the composition of both the gut and lung microbiota. Notably, as lung metastases are among the most severe complications in CRC, berberine has demonstrated protective effects on the human bronchial cell line BEAS-2B after incubation with conditioned media from CRC cell lines HT29 and RKO. These effects are linked to increased E-cadherin and fibronectin expression, contributing to the reversal of indirect tumor cell–induced damage. Additionally, berberine reduced RAD51 upregulation in bronchial cells, suggesting a protective role against metastatic progression [62]. In the same study, mice with colon cancer treated with berberine combined with probiotics exhibited reduced IL-17 and IFN-γ levels and increased IL-10 in lung tissue (Table 5). Compared to probiotics alone, berberine more effectively enriched lung microbiota involved in lysosome metabolism, flavone and flavonol biosynthesis, and glycosphingolipid pathways, and significantly enhanced lung microbiota alpha diversity. Taxonomically, berberine increased *Bacteroidota*, *Bacteroidia*, *Bacteroidales*, *Lactobacillaceae*, *Lactobacillus*, and *Acinetobacter* while reducing *Actinobacteria*, *Bacillales*, *Staphylococcaceae*, and *Staphylococcus*.

In vitro studies using the human CRC cell line HT29 confirmed a dose-dependent inhibitory effect of berberine on cell viability. These pro-apoptotic effects were associated with an increased abundance of *Bacteroidota* and *Proteobacteria* and a decrease in *Ruminococcus*. Berberine also raised serum sodium butyrate levels by inhibiting histone deacetylase type 1 expression [36]. The same study also conducted an in vivo experiment in mice with induced CRC, where both berberine alone and its combination with probiotics led to a reduction in tumor growth. However, the most pronounced effect was observed with the probiotic treatment alone, which significantly inhibited tumor progression.

In another research using murine models of colitis-associated CRC, berberine inhibited tumor growth and reduced Ki-67 expression, a marker of cell proliferation, while improving intestinal dysbiosis by increasing *Akkermansia*, *Lactobacillus*, and *Bacteroides* and modulating tryptophan metabolism and Wnt signaling pathways [60].

Other compounds that emerged during our literature review were hawthorn-derived polysaccharides (HPS), bioactive compounds found in hawthorn fruit. These compounds were shown to ameliorate multiple pathological parameters in mice with experimentally induced CRC. They attenuate inflammation and tissue damage, inhibit tumor growth, and restore body weight and colonic length. These effects are mediated by the induction of apoptosis in neoplastic cells and upregulation of caspase-3 expression in colonic tissue [63], resulting in a significant reduction in tumor number. Moreover, the cytokine profile of HPS-treated animals more closely resembled that of the control group than that of the untreated CRC model, with decreased levels of IL-6, IL-8, IL-17, and IL-1β. Concomitantly, an increase in *Firmicutes* abundance and a decrease in *Bacteroidota* were observed.

Importantly, one major drawback of current chemotherapeutics like 5-fluorouracil is mucosal toxicity. In this way, berberine appears to counteract this limitation by attenuating epithelial damage and modulating pro-inflammatory cytokines in murine models, likely mediated by increasing *Akkermansia* abundance and reducing *Escherichia* and *Shigella*—bacteria with pathogenic potential [61].

**Figure 2:**
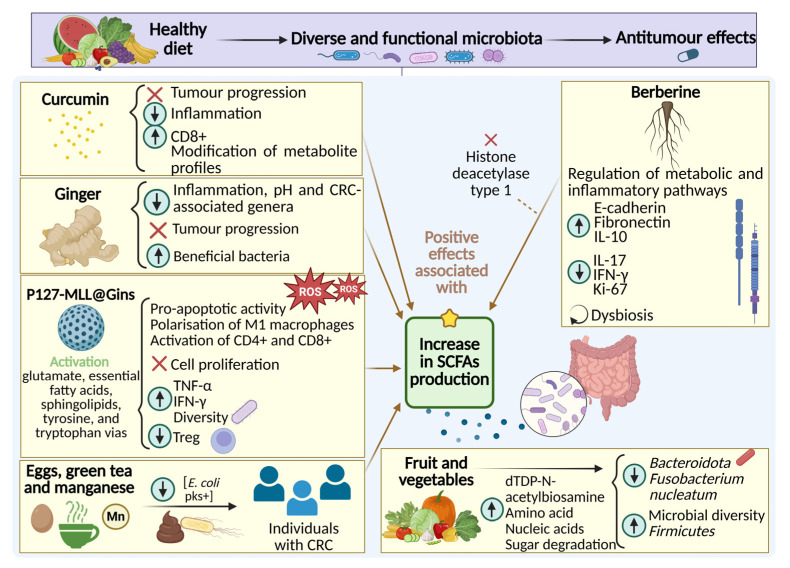
Mechanisms of action of dietary components involved in the modulation of gut microbiota, inflammatory response, and CRC progression. The observed beneficial effects are mainly associated with increased levels of SCFAs.

In summary, the accumulated evidence from in vitro, in vivo, and clinical studies strongly supports the therapeutic potential of some dietary compounds regarding microbiota modulation to reshape gut microbial ecology, reduce inflammation and oxidative stress, and ultimately contribute to improved outcomes in CRC. Further details and a comprehensive analysis of each study mentioned are provided in Table S1 of the Supplementary Material. Moreover, the specific effects of each dietary component are illustrated in Figure 2.

## 4. Discussion

The accumulated evidence in this systematic review reinforces the central concept that gut microbiota modulation represents a mechanistically plausible and nutritionally actionable target for CRC prevention and management. Beyond descriptive associations, the reviewed studies have revealed that diet-derived compounds and microbial interventions converge on shared antitumor pathways, including enhanced epithelial barrier integrity, reduced pro-inflammatory signaling, attenuated oxidative stress, and regulation of host immunometabolism. Comparative analysis across intervention types suggests that prebiotics and bioactive polysaccharides are particularly effective at enriching *Bacillota* and *Bacteroidota* taxa and stimulating SCFA production by genera such as *Roseburia*, *Faecalibacterium*, and *Akkermansia*. These shifts correlate with increased butyrate levels, histone deacetylase inhibition, and pro-apoptotic signaling within colonocytes [25,26,27,28,29]. Although the mechanistic consistency of these findings is notable, most derive from preclinical animal models, limiting their direct clinical extrapolation.

Probiotic strains, including *Lacticaseibacillus rhamnosus*, *L. paracasei*, *Lactobacillus gallinarum*, and *Bifidobacterium bifidum*, demonstrate consistent immunomodulatory activity through competitive exclusion, production of antimicrobial peptides, and regulation of NF-κB and STAT3 via Toll-like receptor (TLR) signaling [33,34,35]. Multi-strain formulations demonstrate broader benefits than single strains, likely due to synergistic metabolite production and enhanced niche complementarity [36,37,38]. Importantly, several clinical studies support the capacity of probiotics and symbiotics to improve epithelial barrier function, reduce mucosal inflammation, and enhance tolerance to chemotherapy and radiotherapy, providing translational relevance beyond preclinical settings [31,45,46].

Postbiotic metabolites such as butyrate, polyhydroxybutyrate, and exopolysaccharides reproduce many immunometabolic benefits of live probiotics without viability constraints, positioning them as promising next-generation biotherapeutics [35,43,47]. Their ability to stimulate IL-10 and IL-12, promote regulatory T cell populations, and suppress pro-inflammatory responses suggests potential applicability in oncology. However, the clinical evidence base remains sparse compared to probiotic interventions.

Dietary bioactives including curcumin, gingerol, and berberine further expand the nutritional toolbox. Curcumin’s dependency on an intact gut microbiota for optimal antitumor efficacy exemplifies a bidirectional pharmacomicrobiomic relationship in which microbial metabolism determines host drug responsiveness [50,51]. Ginger-derived metabolites enhance Lactobacillus and Akkermansia populations, improving anti-inflammatory signaling and contributing to tumor suppression [56,57]. Berberine exerts dual-site effects on gut and lung microbiota while modulating systemic inflammation and butyrate metabolism [36,60,61,62]. Comparative studies indicate that probiotics outperform berberine in tumor suppression and pro-apoptotic signaling, whereas berberine offers complementary metabolic modulation, supporting the rationale for personalized or combined approaches.

Despite these promising outcomes, the current evidence base is fragmented. Most studies are preclinical and employ chemically induced or xenograft CRC models, which only partially replicate the dietary, genetic, and environmental heterogeneity of human disease. Considerable variability in animal species, age, sex, dosing regimens, and microbiota profiling techniques—from classical culture methods to 16S rRNA sequencing and shotgun metagenomics—introduces methodological heterogeneity that hampers comparability. Study endpoints vary widely, with some focusing on metabolic outputs (e.g., SCFAs), others on immune parameters, tumor burden, or microbial taxonomy. This inconsistency precludes robust meta-analysis and limits the capacity to identify universally effective interventions. Furthermore, baseline microbiome characterization is often lacking, despite the well-recognized influence of inter-individual microbial composition on intervention outcomes.

The scarcity of head-to-head clinical trials among prebiotic, probiotic, synbiotic, and postbiotic strategies represents an additional evidence gap, limiting our ability to draw comparative efficacy conclusions. To enhance reproducibility and clinical translation, future research should incorporate harmonized protocols, standardized microbiome analytical pipelines, transparent reporting of dietary intake, and unified microbial and host biomarker panels. Increased inclusion of human cohorts and longitudinal study designs will be essential to establish causal inference and to define patient phenotypes most likely to benefit from microbiota-directed interventions.

Collectively, the emerging clinical findings suggest that microbiota-directed nutrition may evolve into an integral component of multimodal CRC management. By enhancing therapeutic efficacy, mitigating adverse effects, and accelerating recovery, these interventions offer a human-centered, low-toxicity strategy aligned with precision oncology principles. Continued investment in well-controlled clinical trials is essential to clarify optimal formulations, dosing strategies, and synergistic combinations, ultimately facilitating safe and effective integration into routine oncology practice.

## 5. Conclusions

This systematic review provides comprehensive evidence supporting the pivotal role of nutrition-based strategies in shaping the gut microbiota and counteracting the dysbiosis associated with CRC. Across preclinical and clinical settings, prebiotics, probiotics, synbiotics, and postbiotics consistently demonstrate the capacity to enhance microbial equilibrium, modulate immune and inflammatory pathways, and restore epithelial integrity. Likewise, dietary interventions rich in fiber, polyphenols, and bioactive compounds such as berberine exhibit additive benefits by attenuating oxidative stress, chronic inflammation, and metabolic dysregulation—key drivers of CRC pathogenesis.

Comparative data reveal that multi-component interventions, particularly synbiotics combining fermentable fibers and probiotics, achieve superior outcomes compared to single-compound strategies, emphasizing the importance of synergistic approaches. Furthermore, the complementary effects of probiotics and berberine highlight the potential of combined or personalized therapies that leverage both microbial and host mechanisms. However, despite growing mechanistic and translational evidence, most data still originate from preclinical studies or clinical trials in non-CRC populations. The paucity of randomized controlled trials specifically addressing CRC, coupled with the lack of standardized endpoints, remains a critical barrier to clinical implementation. In this context, this systematic review contributes scientifically by consolidating dispersed evidence into a cohesive framework that connects molecular mechanisms, microbiota dynamics, and clinical outcomes. It establishes a conceptual foundation for future precision-nutrition interventions that integrate microbiome modulation into CRC prevention and treatment paradigms.

In conclusion, advancing from preclinical insights to robust, well-designed human studies—supported by multi-omics integration and personalized strategies—will be essential to unlock the full therapeutic potential of microbiota-targeted nutrition. In this way, nutrition science can evolve from a preventive adjunct to a central, evidence-based instrument in reducing CRC incidence, enhancing therapy tolerance, and improving patient survival and quality of life.

## 6. Future Perspectives

The convergence of microbiome science and precision nutrition represents a transformative direction for CRC prevention and therapy. Inter-individual variability in microbiota composition, dietary patterns, and host genetics necessitates a shift from generalized dietary recommendations to tailored, microbiota-informed interventions. Such personalization could optimize response to nutritional therapies while minimizing interindividual variability. Multi-omics integration—combining metagenomics, metabolomics, transcriptomics, and proteomics—will be essential to uncover causal pathways linking diet, microbiota, and tumorigenesis. These approaches may also identify microbial biomarkers predictive of dietary responsiveness, enabling stratified clinical trials. Furthermore, systems biology models could aid in mapping the complex interactions between dietary components, microbial metabolites, and oncogenic signaling pathways.

From a clinical perspective, integrating microbiota-targeted nutrition with conventional treatments may enhance therapeutic precision. By mitigating chemotherapy-induced mucositis, improving drug bioavailability, and modulating systemic inflammation, dietary biotics could serve as metabolic adjuvants to standard therapies. On a public health level, establishing evidence-based dietary frameworks that foster microbial resilience could reduce CRC incidence and recurrence. These future directions position microbiota-centered nutrition not merely as supportive care, but as a scientifically grounded, mechanism-based pillar of integrative oncology.

## Figures and Tables

**Table 1:** Summary of the main findings reported for different prebiotics (PTSO, gingenosides, rice bran and oat bran), classified according to the type of study [25,26,27,28,29,30,31].

Outcome	Prebiotic	Model of CRC Used
↓ CRC cells viability	PTSO Ginsenosides	Cell lines
↑ Microbial diversity	PTSO	
↑ SCFAs		
	Rice bran	Murine
↓ Oxidative stress	PTSO	Cell lines
↑ Beneficial bacteria	Ginsenosides Rice bran	Murine
	Rice bran	Individuals with elevated CRC risk (human)
**↓** White blood cell	Oat bran	Individuals with CRC undergoing radiotherapy (human)
**↓** Inflammation	Ginsenosides	Murine
↓ Tumor growth		
↑ Intestinal barrier integrity		
**↓** Pathogenic species	Rice bran	
**↓** Neoplastic lesions		
**↓** CRC-related metabolites		

**Table 2:** Effects of specific probiotics or combinations of multiple strains, grouped according to the type of study, showing their impact on the gut microbiota and their potential as a therapeutic approach for CRC [25,33,34,35,36,37,39,41,42,43,45,46].

Outcome	Probiotic	Model of CRC Used
↑ Microbial diversity	*Parabacteroides johnsonii* Mix of *Bifidobacterium longum*, *Lactobacillus casei*, *Lactobacillus rhamnosus*, *Streptococcus thermophiles* and *Clostridium butyricum*	Murine
	Mix of *Bifidobacterium infantis*, *Lactobacillus acidophilus*, *Enterococcus faecalis*, and *Bacillus cereus*	Human
**↓** Pathogenic species	Mix of *Lacticaseibacillus* strains	Cell lines
	*Lactobacillus gallinarum* *Clostridium butyricum* Mix of *Bifidobacterium longum*, *Lactobacillus casei*, *Lactobacillus rhamnosus*, *Streptococcus thermophiles* and *Clostridium butyricum*	Murine
	Mix of *Lacticaseibacillus* strains	Human
↑ Beneficial bacterial	*Lactobacillus gallinarum* *Akkermansia muciniphila* *Clostridium butyricum* Mix of *Bifidobacterium longum*, *Lactobacillus casei*, *Lactobacillus rhamnosus*, *Streptococcus thermophiles* and *Clostridium butyricum*	Murine
**↓** Cancer cells	Mix of *Lacticaseibacillus* strains *Clostridium butyricum* Mix of *Bifidobacterium*, *Lactobacillus*, and *Enterococcus*	Cell lines
	Mix of *Bifidobacterium longum*, *Lactobacillus casei*, *Lactobacillus rhamnosus*, *Streptococcus thermophiles* and *Clostridium butyricum*	Murine
↑ SCFAs	Mix of *Lacticaseibacillus* strains	Cell lines
	Mix of *Bifidobacterium bifidum* and *Lactobacillus gasseri* *Clostridium butyricum*	Murine
	Mix of *Lacticaseibacillus* strains Mix of *Bifidobacterium infantis*, *Lactobacillus acidophilus*, *Enterococcus faecalis*, and *Bacillus cereus*	Human
**↓** Inflammation	Mix of *Lacticaseibacillus* strains	Cell lines
	Mix of *Bifidobacterium bifidum* and *Lactobacillus gasseri* *Akkermansia muciniphila* Mix of *Bifidobacterium longum*, *Lactobacillus casei*, *Lactobacillus rhamnosus*, *Streptococcus thermophiles* and *Clostridium butyricum*	Murine
	Mix of *Lacticaseibacillus* strains	Human
**↓** Tumor growth	*Parabacteroides johnsonii* *Clostridium butyricum* Mix of *Bifidobacterium longum*, *Lactobacillus casei*, *Lactobacillus rhamnosus*, *Streptococcus thermophiles* and *Clostridium butyricum* Mix of *Bifidobacterium*, *Lactobacillus*, and *Enterococcus*	Murine
**↓** Number/size of tumors	*Lactobacillus gallinarum* Mix of *Bifidobacterium bifidum* and *Lactobacillus gasseri*	
**↓** Body weight loss	Mix of *Bifidobacterium bifidum* and *Lactobacillus gasseri* *Akkermansia muciniphila*	
**↓** Symptoms	*Akkermansia muciniphila* *Bifidobacterium bifidum* and *Lactobacillus gasseri* Mix of *Bifidobacterium longum*, *Lactobacillus casei*, *Lactobacillus rhamnosus*, *Streptococcus thermophiles* and *Clostridium butyricum* Mix of *Bifidobacterium infantis*, *Lactobacillus acidophilus*, *Enterococcus faecalis*, and *Bacillus cereus*	
↑ Intestinal barrier integrity	*Bifidobacterium animalis* ssp. *lactis* BX-245	
*↑ Immune mediators*		

**Table 3:** This table illustrates the effects associated with metabolites produced by various bacterial species. It underscores that research focusing on these postbiotics in CRC models remains relatively limited, despite their pronounced effects on both CRC pathology parameters and gut microbiota composition, which are comparable to those observed with probiotics [35,43].

Outcome	Metabolites from	Model of CRC Used
**↓** Cancer cells	*Lactobacillus gallinarum*	Cell lines
		CRC organoids (from humans)
↑ SCFAs	PHB	Murine
	Mix of *Lactobacillus paracasei* SD1 and *Lacticaseibacillus rhamnosus* SD11	Human
↑ Microbial diversity	PHB	Murine
**↓** Pathogenic species	Mix of *Lactobacillus paracasei* SD1 and *Lacticaseibacillus rhamnosus* SD11	Human
**↓** Inflammation	PHB	Murine
	Mix of *Lactobacillus paracasei* SD1 and *Lacticaseibacillus rhamnosus* SD11	Human
**↓** Number/size of tumors	PHB	Murine

**Table 5:** This table presents the results of various combinations of microbiota-modulating strategies used as potential treatments for CRC. It highlights that combining different approaches may provide greater benefits than using each strategy individually [31,36,37,62].

Outcome	Combination	Model of CRC Used
**↓** White blood cell	Oat bran + *Lactobacillus plantarum* HEAL19 + blueberry husks	Individuals with CRC undergoing radiotherapy (human)
**↓** Inflammation		
**↓** Tissue damage		
↑ Microbial diversity		
**↓** CRC development	Quercetine + *Bifidobacterium bifidum* and *Lactobacillus gasseri*	Murine
**Benefits derived from the combination**		
**↓** Inflammation	Berberine and probiotics (comparison with their separate use	Murine
↑ Microbial diversity		
↑ Beneficial bacteria		
**↓** Pathogenic species		

## Data Availability

No new data were created or analyzed in this study. Data sharing is not applicable to this article.

## Supplementary Materials

The following supporting information can be downloaded at: https://www.mdpi.com/article/10.3390/nu17223565/s1, Prisma Checklist. PRISMA 2020 checklist for systematic reviews, prepared according to the Preferred Reporting Items for Systematic Reviews and Meta-Analyses guidelines [64]. Table S1. Summary of the studies included in the review, specifying experimental design, model or sample, type of intervention, and key findings.

## Abbreviations

The following abbreviations are used in this manuscript:

CRC	Colorectal Cancer
FMT	Fecal Microbiota Transplantation
hBD	human β-Defensin
HPS	Hawthorn-derived Polysaccharides
PHB	Polyhydroxybutyrate
PRISMA	Preferred Reporting Items for Systematic Reviews and Meta-Analyses
PTSO	Propyl Propane Thiosulfonate
SCFAS	Short-Chain Fatty Acids
