# Supplementary material for: Modulating Strategies of the Intestinal Microbiota in Colorectal Cancer
Source: Nutrients. 2025 Nov 14;17(22):3565. doi: 10.3390/nu17223565 (PMC12655525; doi:10.3390/nu17223565)
Supplement: Supplementary file 1 [file nutrients-17-03565-s001.zip › nutrients-3936936-supplementary/SUPPLEMENTARY.pdf]

PRISMA 2020 Checklist

Section and Topic	Item #	Checklist item	Location where item is reported
**TITLE**			
Title	1	Identify the report as a systematic review.	Pag. 1
**ABSTRACT**			
Abstract	2	See the PRISMA 2020 for Abstracts checklist.	Pag. 1-2
**INTRODUCTION**			
Rationale	3	Describe the rationale for the review in the context of existing knowledge.	Pag. 3-4
Objectives	4	Provide an explicit statement of the objective(s) or question(s) the review addresses.	Pag. 4-5
**METHODS**			
Eligibility criteria	5	Specify the inclusion and exclusion criteria for the review and how studies were grouped for the syntheses.	Pag. 6
Information sources	6	Specify all databases, registers, websites, organisations, reference lists and other sources searched or consulted to identify studies. Specify the date when each source was last searched or consulted.	Pag. 5 and Fig. 1
Search strategy	7	Present the full search strategies for all databases, registers and websites, including any filters and limits used.	Pag. 5 and Fig. 1
Selection process	8	Specify the methods used to decide whether a study met the inclusion criteria of the review, including how many reviewers screened each record and each report retrieved, whether they worked independently, and if applicable, details of automation tools used in the process.	Pag. 6-7
Data collection process	9	Specify the methods used to collect data from reports, including how many reviewers collected data from each report, whether they worked independently, any processes for obtaining or confirming data from study investigators, and if applicable, details of automation tools used in the process.	Pag. 6-7
Data items	10a	List and define all outcomes for which data were sought. Specify whether all results that were compatible with each outcome domain in each study were sought (e.g. for all measures, time points, analyses), and if not, the methods used to decide which results to collect.	Pag. 6-7
	10b	List and define all other variables for which data were sought (e.g. participant and intervention characteristics, funding sources). Describe any assumptions made about any missing or unclear information.	Pag. 6-7
Study risk of bias assessment	11	Specify the methods used to assess risk of bias in the included studies, including details of the tool(s) used, how many reviewers assessed each study and whether they worked independently, and if applicable, details of automation tools used in the process.	Pag. 7
Effect measures	12	Specify for each outcome the effect measure(s) (e.g. risk ratio, mean difference) used in the synthesis or presentation of results.	Pag. 6-7
Synthesis methods	13a	Describe the processes used to decide which studies were eligible for each synthesis (e.g. tabulating the study intervention characteristics and comparing against the planned groups for each synthesis (item #5)).	Pag. 6-7
	13b	Describe any methods required to prepare the data for presentation or synthesis, such as handling of missing summary statistics, or data conversions.	Pag. 6-7
	13c	Describe any methods used to tabulate or visually display results of individual studies and syntheses.	Pag. 6-7
	13d	Describe any methods used to synthesize results and provide a rationale for the choice(s). If meta-analysis was performed, describe the model(s), method(s) to identify the presence and extent of statistical heterogeneity, and software package(s) used.	Not applicable
	13e	Describe any methods used to explore possible causes of heterogeneity among study results (e.g. subgroup analysis, meta-regression).	Not applicable
	13f	Describe any sensitivity analyses conducted to assess robustness of the synthesized results.	Not applicable
Reporting bias assessment	14	Describe any methods used to assess risk of bias due to missing results in a synthesis (arising from reporting biases).	Not applicable
Certainty	15	Describe any methods used to assess certainty (or confidence) in the body of evidence for an outcome.	Not applicable

Section and Topic	Item #	Checklist item	Location where item is reported
assessment			
**RESULTS**			
Study selection	16a	Describe the results of the search and selection process, from the number of records identified in the search to the number of studies included in the review, ideally using a flow diagram.	Fig.1 and Pag. 7
	16b	Cite studies that might appear to meet the inclusion criteria, but which were excluded, and explain why they were excluded.	Fig.1 and Pag. 7
Study characteristics	17	Cite each included study and present its characteristics.	Pag. 8-23
Risk of bias in studies	18	Present assessments of risk of bias for each included study.	Not applicable
Results of individual studies	19	For all outcomes, present, for each study: (a) summary statistics for each group (where appropriate) and (b) an effect estimate and its precision (e.g. confidence/credible interval), ideally using structured tables or plots.	Table 1 and Pag. 8-23
Results of syntheses	20a	For each synthesis, briefly summarise the characteristics and risk of bias among contributing studies.	Not applicable
	20b	Present results of all statistical syntheses conducted. If meta-analysis was done, present for each the summary estimate and its precision (e.g. confidence/credible interval) and measures of statistical heterogeneity. If comparing groups, describe the direction of the effect.	Not applicable
	20c	Present results of all investigations of possible causes of heterogeneity among study results.	Table 1 and Pag. 8-23
	20d	Present results of all sensitivity analyses conducted to assess the robustness of the synthesized results.	Not applicable
Reporting biases	21	Present assessments of risk of bias due to missing results (arising from reporting biases) for each synthesis assessed.	Not applicable
Certainty of evidence	22	Present assessments of certainty (or confidence) in the body of evidence for each outcome assessed.	Table 1 and Pag. 8-23
**DISCUSSION**			
Discussion	23a	Provide a general interpretation of the results in the context of other evidence.	Pag.24-25
	23b	Discuss any limitations of the evidence included in the review.	Pag.24-25
	23c	Discuss any limitations of the review processes used.	Pag.24-25
	23d	Discuss implications of the results for practice, policy, and future research.	Pag. 26-27
**OTHER INFORMATION**			
Registration and protocol	24a	Provide registration information for the review, including register name and registration number, or state that the review was not registered.	Not applicable
	24b	Indicate where the review protocol can be accessed, or state that a protocol was not prepared.	Not applicable
	24c	Describe and explain any amendments to information provided at registration or in the protocol.	Not applicable
Support	25	Describe sources of financial or non-financial support for the review, and the role of the funders or sponsors in the review.	Pag. 28-29
Competing interests	26	Declare any competing interests of review authors.	Pag. 29 and Supplementary material
Availability of data, code and	27	Report which of the following are publicly available and where they can be found: template data collection forms; data extracted from included studies; data used for all analyses; analytic code; any other materials used in the review.	Not applicable

PRISMA 2020 Checklist

Section and Topic	Item #	Checklist item	Location where item is reported
other materials			

From: Page MJ, McKenzie JE, Bossuyt PM, Boutron I, Hoffmann TC, Mulrow CD, et al. The PRISMA 2020 statement: an updated guideline for reporting systematic reviews. BMJ 2021;372:n71. doi: 10.1136/bmj.n71. This work is licensed under CC BY 4.0. To view a copy of this license, visit https://creativecommons.org/licenses/by/4.0/

Supplementary Material

Study design	Model used	Type of intervention	Effect observed
In vitro [1]	CRC cell lines: HT29 and T84	Administration of the prebiotic PTSO	Selective and dose-dependent cytotoxicity, increased microbial diversity, enhanced SCFAs production, and mitigation of oxidative stress
In vitro [2]	CRC cell lines: HCT-116, derived from humans, and CT26, derived from mice	Ginsenosides such as 20R-Rg3 and Rg5-BG acting as prebiotics	Induction of apoptosis in tumour cells by activating the caspase-3/Bax/Bcl-2 signalling pathway and modulation of microbiota
In vivo [3]	Rats with colitis-associated CRC (induced model) (n = 10-12 per group)	Use of rice bran as a prebiotic	Increase in the abundance of beneficial bacteria and SCFAs and reduction in pathogenic species, as well as reversing the decrease in goblet cells and improving the thickness of the mucosa in the colon
In vivo [4]	Germ-free mice with induced CRC, transplanted with fecal microbiota from CRC survivors who consumed rice bran daily (n = 3-10 per group)	Prebiotic intake (rice bran) in humans and FMT in a CRC mouse model	Reduction of neoplastic lesions with increased abundance of beneficial bacteria such as *Flavonifractor* and *Oscillibacter*, higher levels of anticancer metabolites like myristoylcarnitine, and decreased presence of tumor-associated species like *Parabacteroides distasonis* and CRC-related metabolites such as tartrate and trimethylamine N-oxide<br>Reduced proinflammatory metabolites and antioxidant and anti-inflammatory effects through regulation of key metabolic pathways
In vivo [5]	CRC-induced mice (pseudo germ-free model) receiving FMT from Rh4-treated, CRC-induced donor mice. The sample size was not specified	Use of ginsenoside Rh4 as a prebiotic, in donors, and FMT in recipients	Inhibition of CRC by promoting the growth of *Akkermansia muciniphila* and increasing gut microbiota diversity, restoring intestinal barrier integrity, and reducing inflammatory cytokines and mediators
Clinical pilot study [6]	Chinese adults with elevated CRC risk (n = 19-20 per group)	Dietary intervention with rice bran prebiotic	Significant increase in beneficial taxa: *Firmicutes*, *Lactobacillus*, *Prevotella*, *Lactobacillales*, *Bifidobacteria*, and the *Firmicutes/Bacteroidota* ratio
Clinical study [7]	Patients with CRC undergoing	Administration of the prebiotic oat bran	Attenuation of white blood cell count in both interventions, while in the synbiotic group

	radiotherapy (n = 10 per group)	alone and its combined use with the probiotic *Lactobacillus plantarum* HEAL19 and blueberry husks	reduced inflammation and fibrosis were observed, along with better preservation of mucosa-associated microbiota diversity
In vitro [8]	Human CRC cell lines: Caco-2 and HIEC-6	Use of *Lacticaseibacillus* strains as probiotics	Inhibition of pathogen-related CRC, stimulation of antimicrobial peptides, and suppression of cancer cells through the production of SCFAs, especially butyrate, by probiotic strains such as *L. paracasei* SD1 and *L. rhamnosus* SD11, which also induce IL-10 and reduce IL-8
In vitro [9]	Human CRC cell lines: Caco-2 and HIEC-6	Use of *Lacticaseibacillus paracasei* SD1, *Lacticaseibacillus rhamnosus* SD4, *Lacticaseibacillus rhamnosus* SD11, and *Lacticaseibacillus rhamnosus* GG as probiotics and their culture supernatants free of cells	Strain-specific effects were observed between probiotic cells and their cell-free supernatants, with *L. rhamnosus* SD11 showing the strongest inhibition of pathogen growth. Overall, a reduction in pro-inflammatory cytokines was reported. Notably, *L. paracasei SD1* and *L. rhamnosus* SD11 significantly increased the expression of antimicrobial peptides such as hBD and anti-inflammatory cytokines like IL-10. Both strains also suppressed cell proliferation, particularly in Caco-2 cells
In vitro, in vivo and *ex vivo* study (human organoids) [10]	Human CRC cells (HCT116 and LoVo cell lines), as well as normal colon epithelial cells (NCM460 cell line), murine models of colitis-associated CRC (n = 5-10 per group), and human organoids from two patients	Use of *Lactobacillus gallinarum,* both directly as a probiotic and through the postbiotics it produces	*In vivo* results demonstrated an antitumor effect, evidenced by a reduction in both the number and size of tumors, accompanied by an increase in beneficial bacterial populations and a decrease in pathogenic species. *In vitro*, the culture supernatant of *Lactobacillus gallinarum* inhibited tumor cell proliferation and selectively induced apoptosis in cancer cells, sparing normal colon epithelial cells. These antitumor effects were associated with elevated levels of indole-3-lactic acid. A pro-apoptotic effect was also observed in patient-derived organoids
In vivo [11]	Transgenic mice harboring the ApcMin/+ mutation, commonly used to study CRC progression (n = 12 per group)	Standard diet supplemented with microencapsulated probiotics (*Bifidobacterium bifidum* and *Lactobacillus gasseri*) and quercetin; compared to standard diet alone and standard diet with	Both probiotic-based interventions resulted in a mitigation of body weight loss, intestinal bleeding, and tumour burden in ApcMin/+ mice. The combination of *Bifidobacterium bifidum* and *Lactobacillus gasseri* with quercetin led to the most pronounced reduction in aberrant crypt foci and adenomas, an effect linked to the downregulation of the canonical Wnt/β-catenin signalling pathway. Nonetheless, probiotic supplementation

		probiotics only	without quercetin also produced favourable antitumour effects
In vivo [12]	The study used two established murine models of CRC: an orthotopic model with MC-38 cell injection into the cecum, and an inflammation-driven model induced by AOM/DS. The sample size was not specified	Administration of *Bifidobacteria* probiotics, either alone or in combination with *Lactobacilli*	The combination of both probiotic strains improved tumor burden in the MC-38 model by modulating gut microbiota composition and promoting SCFAs production. In contrast, the exclusive administration of *Bifidobacteria* proved optimal in the AOM/DSS model, improving inflammation and exerting a protective effect
In vivo [13]	Study with CRC-induced mouse model (n = 7-8 per group)	Probiotic administration of *Parabacteroides johnsonii* and *Lactococcus formosensis*	Inhibition of tumor growth, improvement of histopathological parameters, restoration of gut microbial diversity, notably increasing beneficial species such as *Bifidobacterium pseudolongum* and *Lactobacillus,* and modulation of amino sugar metabolism and tryptophan biosynthesis relevant to tumor microenvironment regulation
In vivo [14]	Mice with induced CRC (n = 6 for each group)	Probiotic administration of *Akkermansia muciniphila*	Attenuation of weight loss, symptoms, and intestinal inflammation through modulation of the NF-κB pathway, induction of apoptosis, and reshaping of microbial communities, promoting beneficial taxa such as *Muribaculaceae* and reducing pro-inflammatory species like *Bacteroides* and *Parasutterella*
In vivo and *in vitro* [15]	Mice with induced CRC (Apcmin/+) (n = 10 per group) and human CRC cell lines including HCT116, Caco-2, and HCT8 for *in vitro* studies	Probiotic administration of *Clostridium butyricum*	CRC development was inhibited in the animal model, with reduced proliferation and pro-apoptotic effects observed *in vitro*. These effects were associated with suppression of the Wnt/β-catenin pathway and modulation of gut microbiota composition, promoting SCFAs production and beneficial bacteria, while decreasing pathogenic species
In vivo [16]	Mice with induced CRC (n = 5 for each group)	Administration of probiotic fermented milk containing *Bifidobacterium animalis* ssp. *lactis* BX-245	Increased tumour microenvironment levels, improved intestinal barrier integrity and increased systemic immune mediators such as IL-2 and IFN-γ
Clinical study [17]	30 patients with grade II–III CRC, post-curative	Supplementation with *Lactobacillus paracasei* SD1 and	Promotion of the growth of SCFAs-producing bacteria and reduction of pathogenic species, as well as increasing levels of anti-

	resection, without therapy (n = 10 per group)	*Lacticaseibacillus rhamnosus* SD11 and their metabolites	inflammatory cytokines such as IL-10 and IL-12
In vivo [18]	Female CRC model mice (n = 24, divided into four groups)	Probiotics, either alone or encapsulated in resistant starch, were administered to mice treated with 5-fluorouracil. Additional groups included mice receiving only 5-FU and untreated controls	The combination of chemotherapy with resistant-starch-encapsulated probiotics inhibited tumor growth and promoted pro-apoptotic activity. In addition, both this group and the one that received chemotherapy with non-encapsulated probiotics reversed the inflammation and NF-κB signaling activation induced by the chemotherapeutic agent. Furthermore, both probiotic-treated groups showed a protective effect against chemotherapy-induced gut dysbiosis
Clinical study [19]	CRC patients (n = 100)	Administration of a probiotic cocktail containing *Bifidobacterium infantis, Lactobacillus acidophilus, Enterococcus faecalis,* and *Bacillus cereus*	Restoration of microbial diversity altered by chemotherapy and increase in SCFAs-producing taxa, alleviating treatment-related gastrointestinal symptoms such as diarrhoea and abdominal distension
In vivo [20]	Rats with induced CRC (n = 8 per group with CRC induction, plus 2 controls per cohort)	Supplementation of PHB as a postbiotic	Regulation of the composition and diversity of the microbiota to a healthy state and increased production of SCFAs, leading to a reduction in tumor area and number, as well as inflammation
Clinical study [21]	Healthy individuals of Japanese origin (n = 223)	Dietary intervention to assess fecal E. coli pks+ concentration, related to CRC development	Concentrations of E. coli pks+ in fecal samples were negatively correlated with green tea and manganese intake
Clinical pilot study [22]	CRC survivors (n = 28)	A cross-sectional observational study assessing dietary and physical activity habits in relation to the American Cancer Society guidelines, with particular emphasis on fruit and vegetable intake	There is a positive relationship between fruit and vegetable consumption and increased gut microbial diversity, along with a higher abundance of *Firmicutes* and a reduction in *Bacteroidota*. These effects are mediated by changes in gene expression and multiple metabolic pathways, including the activation of pathways involved in the production of SCFAs
In vivo and *in vitro* [23]	CRC cells (CT26, from mice, and RKO, human) and mice as a model of the	Oral administration of curcumin and FMT	Enhanced microbial diversity and improved gut microbiota composition, with increased abundance of *Lactobacillus* and *Kineothrix*. These changes are associated with higher

	disease (n =.5-7 per group)		intratumoral infiltration of CD8+ T cells, induction of ferroptosis, and promotion of apoptosis in tumor cells. The antitumor effects can be transferred through FMT and are abolished when gut microbiota is depleted by antibiotic treatment
In vivo [24]	Mice with induced CRC (n = 10 per group)	Oral administration of curcumin	A recovery of colon length and architecture was observed, along with a marked reduction in tumor development. These effects were associated with an increase in beneficial bacteria such as Clostridia_UCG-014, *Bifidobacterium*, and *Lactobacillus*, and a decrease in harmful bacteria including *Ileibacterium*, *Monoglobus*, and *Desulfovibrio*, which were highly elevated in cancer-bearing mice compared to the control group
In vitro and *in vivo* [25]	CRC cells (HCT-116 and HT-29) and mouse model (n = 3-4 per group)	Administration of curcumin and a fraction rich in tocopherols from vitamin E	*In vitro* reduction of tumor cell proliferation and *in vivo* suppression of HCT-116-derived xenograft growth, associated with a significant increase in *Lactobacillaceae*, *Bifidobacteriaceae*, and *Clostridium* cluster XIVa, contributing to an anti-inflammatory environment. Likewise, a decrease in the relative abundance of the *Bacteroidota* and *Firmicutes* phyla was observed
In vitro [26]	Human CRC cell lines: LoVo and HCT116	Administration of nanoparticles containing zingerone and free zingerone, derived from ginger, in human CRC cells	Both forms of administration showed cytotoxicity, pro-apoptotic effects, and inhibition of tumor cell colony formation, with a stronger effect observed for the nanoparticles. The nanoparticle effect was associated with high activation of caspase-3 and PARP, increased p21 expression, inhibition of CDC25C, CDK1, and Cyclin B, and reduced levels of Cyclin D, CDK2, and Cyclin A
In vitro [27]	CRC cell line: HT-29	Administration of ginger capsules	Potent cytotoxic activity of ginger against CRC cells linked to its high quercetin content
In vitro [28]	Mouse fecal inoculum	*In vitro* model simulating the digestive and fermentative processes of ginger	Alteration of the fecal microbiota structure, favoring the growth of beneficial bacterial populations such as *Bifidobacterium* and *Enterococcus*, along with an increase in SCFAs and a corresponding decrease in pH
Clinical study [29]	Healthy participants (n= 123)	Crossover intervention with participants consuming fresh ginger juice	Increased microbial species richness and shifts in key bacterial ratios, including a decreased *Prevotella–Bacteroides* ratio and reduced pro-inflammatory *Ruminococcus*.

			Trends also showed higher *Firmicutes–Bacteroidota* ratio, increased *Proteobacteria*, and elevated anti-inflammatory *Faecalibacterium*
In vivo [30]	Mice with induced CRC (n = 6 per group)	Oral administration of the P127-MLL@Gins nanoparticle, followed by exposure to alternating magnetic fields	Treatment with the P127-MLL@Gins nanoparticle significantly reduced tumor number and size, induced apoptosis in cancer cells, and activated the immune response, including increased CD4+ and CD8+ T cells, M1 macrophages, and pro-inflammatory cytokines. It also improved gut microbiota composition by increasing diversity and the *Firmicutes/Bacteroidota* ratio, enhancing the abundance of beneficial bacteria such as *Bacillus*, and reducing harmful genera like *Alloprevotella* and *Bacteroides*. Additionally, it stimulated metabolic pathways related to essential amino acids and lipids
Clinical study [31]	Adults previously diagnosed with colorectal adenoma (n = 68)	Oral ginger or placebo supplementation for 6 weeks	Significant reduction in the relative abundance of CRC-associated genera such as *Akkermansia, Bacteroides,* and *Ruminococcus* following ginger supplementation. No notable changes were observed in overall gut microbial diversity
In vitro and *in vivo* [32]	CRC cell lines (HT29 and RKO), along with the human bronchial epithelial cell line BEAS-2B, were used for the *in vitro* studies. Mice with CRC-induced tumors served as the *in vivo* model (n = 8-9 per group)	In the *in vitro* experiments, BEAS-2B cells were incubated with berberine and conditioned media from HT29 and RKO cells. For the *in vivo* studies, mice were treated with berberine and/or probiotics	Increased expression of E-cadherin and fibronectin helped reverse tumor cell–induced damage and reduced RAD51 overexpression in bronchial cells, suggesting a protective role of berberine against metastatic progression.<br>*In vivo* results showed that combining berberine with probiotics lowered IL-17 and IFN-γ levels and increased IL-10 in lung tissue. Compared to probiotics alone, berberine alone more effectively enriched the lung microbiota involved in lysosome metabolism, flavone and flavonol biosynthesis, and glycosphingolipid pathways, significantly enhancing alpha diversity. Berberine also increased *Bacteroidota, Bacteroidia, Bacteroidales, Lactobacillaceae, Lactobacillus,* and *Acinetobacter*, while reducing *Actinobacteria*, *Bacillales*, *Staphylococcaceae*, and *Staphylococcus*
In vitro and *in vivo* [33]	Human CRC cell line HT29 for *in vitro* study and mice with CRC induced for *in*	*In vitro*, treatments with berberine and probiotics were administered. *In vivo*,	Berberine exhibited a dose-dependent inhibition of HT29 cell proliferation, while the probiotic supernatant showed the most significant antiproliferative effect. Both

	vivo study (n = 8-9 per group)	mice received berberine, probiotics, or a combination of both	treatments also reduced HDAC1 mRNA expression. *In vivo*, probiotic treatment significantly inhibited tumor growth in mice, while berberine alone showed moderate effects. Additionally, both treatments modulated gut microbiota composition: berberine increased *Bacteroidota* and *Proteobacteria* and reduced *Ruminococcus*, while probiotics decreased *Verrucomicrobia* and *Akkermansia*. The combination treatment also enhanced microbial diversity and amplified the beneficial shifts in microbiota composition
In vivo [34]	Mice with induced colitis-associated CRC (n = 12 per group)	Oral administration of berberine	Berberine inhibited tumor growth and reduced Ki-67 expression, a marker of cell proliferation, while improving intestinal dysbiosis by increasing *Akkermansia, Lactobacillus,* and *Bacteroides* and modulating tryptophan metabolism and Wnt signaling pathways
In vivo [35]	Mice with induced CRC (n = 12 per group)	Oral administration of HPS	Mitigation of inflammation and tissue damage, inhibition of tumor growth, and restoration of body weight and colonic length. These effects are mediated by the induction of apoptosis in neoplastic cells and the upregulation of caspase-3 expression in colonic tissue, along with the reduction of cytokines IL-6, IL-8, IL-17, and IL-1β, and an increase in *Firmicutes* abundance accompanied by a decrease in *Bacteroidota*
In vitro and *in vivo* [36]	Mice model of mucositis induced by the chemotherapy drug 5-fluorouracil (n = 4-10 per group), CRC cells (SW480) and human intestinal epithelial cells (CCD841)	Administration of oral 5-fluorouracil and berberine	Berberine appears to mitigate 5-fluorouracil-induced mucosal toxicity by attenuating epithelial damage and modulating the pro-inflammatory cytokine profile in mice. These effects are associated with an increased abundance of *Akkermansia* and a decreased prevalence of *Escherichia* and *Shigella*, bacteria with pathogenic potential

Table S1. Summary of the studies included in the review, specifying experimental design, model or sample, type of intervention, and key findings.

REFERENCES

1. Guillamón E, Navajas-Porras B, Delgado-Osorio A, Gil-Martínez L, Mut-Salud N, Cuberos-Escobar A, et al. Evaluation of PTSO delivery approaches for gut microbiota modulation in colorectal cancer: A comparative study of microcapsules containing Allium derivatives. Journal of Functional Foods. 2025;128:106818.
2. Yu P, Xu W, Li Y, Xie Z, Shao S, Liu J, et al. Ginsenosides 20R-Rg3 and Rg5 enriched black ginseng inhibits colorectal cancer tumor growth by activating the Akt/Bax/caspase-3 pathway and modulating gut microbiota in mice. Current Research in Food Science. 2025;10:100978.
3. Tajasuwan L, Kettawan A, Rungruang T, Wunjuntuk K, Prombutara P. Role of Dietary Defatted Rice Bran in the Modulation of Gut Microbiota in AOM/DSS-Induced Colitis-Associated Colorectal Cancer Rat Model. 2023;15(6):1528.
4. Parker KD, Maurya AK, Ibrahim H, Rao S, Hove PR, Kumar D, et al. Dietary Rice Bran-Modified Human Gut Microbial Consortia Confers Protection against Colon Carcinogenesis Following Fecal Transfaunation. 2021;9(2):144.
5. Bai X, Duan Z, Deng J, Zhang Z, Fu R, Zhu C, et al. Ginsenoside Rh4 inhibits colorectal cancer via the modulation of gut microbiota-mediated bile acid metabolism. Journal of advanced research. 2025;72:37-52.
6. So WKW, Chan JYW, Law BMH, Choi KC, Ching JYL, Chan KL, et al. Effects of a Rice Bran Dietary Intervention on the Composition of the Intestinal Microbiota of Adults with a High Risk of Colorectal Cancer: A Pilot Randomised-Controlled Trial. Nutrients. 2021;13(2).
7. Stene C, Xu J, Fallone de Andrade S, Palmquist I, Molin G, Ahrné S, et al. Synbiotics protected radiation-induced tissue damage in rectal cancer patients: A controlled trial. Clinical nutrition (Edinburgh, Scotland). 2025;49:33-41.
8. Thananimit S, Pahumunto N, Teanpaisan R. Characterization of Short Chain Fatty Acids Produced by Selected Potential Probiotic Lactobacillus Strains. Biomolecules. 2022;12(12).
9. Pahumunto N, Teanpaisan R. Anti-cancer Properties of Potential Probiotics and Their Cell-free Supernatants for the Prevention of Colorectal Cancer: an In Vitro Study. Probiotics and antimicrobial proteins. 2023;15(5):1137-50.
10. Sugimura N, Li Q, Chu ESH, Lau HCH, Fong W, Liu W, et al. Lactobacillus gallinarum modulates the gut microbiota and produces anti-cancer metabolites to protect against colorectal tumourigenesis. Gut. 2021;71(10):2011-21.
11. Benito I, Encío IJ, Milagro FI, Alfaro M, Martínez-Peñuela A, Barajas M, et al. Microencapsulated Bifidobacterium bifidum and Lactobacillus gasseri in Combination with Quercetin Inhibit Colorectal Cancer Development in Apc(Min/+) Mice. International journal of molecular sciences. 2021;22(9).
12. Niechcial A, Schwarzfischer M, Wawrzyniak P, Determann M, Pöhlmann D, Wawrzyniak M, et al. Probiotic Administration Modulates Gut Microbiota and Suppresses Tumor Growth in Murine Models of Colorectal Cancer. International journal of molecular sciences. 2025;26(9).
13. Liu J, Zhang Y, Xu L, Gu G, Dong Z. Parabacteroides johnsonii inhibits the onset and progression of colorectal cancer by modulating the gut microbiota. Journal of translational medicine. 2025;23(1):734.
14. Ma X, LvjunYan, Yu X, Guo H, He Y, Wen S, et al. The alleviating effect of Akkermansia muciniphila PROBIO on AOM/DSS-induced colorectal cancer in mice and its regulatory effect on gut microbiota. Journal of Functional Foods. 2024;114:106091.
15. Chen D, Jin D, Huang S, Wu J, Xu M, Liu T, et al. Clostridium butyricum, a butyrate-producing probiotic, inhibits intestinal tumor development through modulating Wnt signaling and gut microbiota. Cancer letters. 2020;469:456-67.

16. Yang C, Zha M, Li L, Qiao J, Kwok LY, Wang D, et al. Bifidobacterium animalis ssp. lactis BX-245-fermented milk alleviates tumor burden in mice with colorectal cancer. Journal of dairy science. 2025;108(2):1211-26.
17. Wanitsuwan W, Pahumunto N, Surachat K, Thananimit S, Wonglapsuwan M, Laohawiriyakamol S, et al. Comparison of the effects of postbiotics and live-probiotics containing Lacticaseibacillus paracasei SD1 and Lacticaseibacillus rhamnosus SD11 in patients with previous colorectal cancer: A randomized controlled trial. Journal of Functional Foods. 2024;123:106576.
18. Wang JL, Chen YS, Huang KC, Yeh CH, Chen MC, Wu LS, et al. Resistant Starch-Encapsulated Probiotics Attenuate Colorectal Cancer Cachexia and 5-Fluorouracil-Induced Microbial Dysbiosis. Biomedicines. 2024;12(7).
19. Huang F, Li S, Chen W, Han Y, Yao Y, Yang L, et al. Postoperative Probiotics Administration Attenuates Gastrointestinal Complications and Gut Microbiota Dysbiosis Caused by Chemotherapy in Colorectal Cancer Patients. Nutrients. 2023;15(2).
20. Fernández J, Saettone P, Franchini MC, Villar CJ, Lombó F. Antitumor bioactivity and gut microbiota modulation of polyhydroxybutyrate (PHB) in a rat animal model for colorectal cancer. International journal of biological macromolecules. 2022;203:638-49.
21. Watanabe D, Murakami H, Ohno H, Tanisawa K, Konishi K, Tsunematsu Y, et al. Association between dietary intake and the prevalence of tumourigenic bacteria in the gut microbiota of middle-aged Japanese adults. Scientific reports. 2020;10(1):15221.
22. Kyaw TS, Upadhyay V, Tolstykh I, Van Loon K, Laffan A, Stanfield D, et al. Variety of Fruit and Vegetables and Alcohol Intake are Associated with Gut Microbial Species and Gene Abundance in Colorectal Cancer Survivors. The American journal of clinical nutrition. 2023;118(3):518-29.
23. Zhou H, Zhuang Y, Liang Y, Chen H, Qiu W, Xu H, et al. Curcumin exerts anti-tumor activity in colorectal cancer via gut microbiota-mediated CD8(+) T Cell tumor infiltration and ferroptosis. Food & function. 2025;16(9):3671-93.
24. Deng W, Xiong X, Lu M, Huang S, Luo Y, Wang Y, et al. Curcumin suppresses colorectal tumorigenesis through restoring the gut microbiota and metabolites. BMC cancer. 2024;24(1):1141.
25. Farhana L, Sarkar S, Nangia-Makker P, Yu Y, Khosla P, Levi E, et al. Natural agents inhibit colon cancer cell proliferation and alter microbial diversity in mice. PLoS One. 2020;15(3):e0229823.
26. Wang JH, Chen YW, Hsieh S, Kung ML. Nanosized Ginger-Derived Phenolic Zingerone Obstructs Cell Cycle G2/M Progression and Initiates Apoptosis in Human Colorectal Cancer. Environmental toxicology. 2025;40(8):1059-71.
27. Al Azzam KM, Al-Areer NW, Al Omari RH, Al-Deeb I, Bounoua N, Negim ES, et al. Assessment of the anticancer potential of certain phenolic and flavonoid components in ginger capsules using colorectal cancer cell lines coupled with quantitative analysis. Biomedical chromatography : BMC. 2024;38(10):e5993.
28. Wang J, Chen Y, Hu X, Feng F, Cai L, Chen F. Assessing the Effects of Ginger Extract on Polyphenol Profiles and the Subsequent Impact on the Fecal Microbiota by Simulating Digestion and Fermentation In Vitro. Nutrients. 2020;12(10).
29. Wang X, Zhang D, Jiang H, Zhang S, Pang X, Gao S, et al. Gut Microbiota Variation With Short-Term Intake of Ginger Juice on Human Health. Frontiers in microbiology. 2020;11:576061.
30. Li B, Zu M, Jiang A, Cao Y, Wu J, Shahbazi MA, et al. Magnetic natural lipid nanoparticles for oral treatment of colorectal cancer through potentiated antitumor immunity and microbiota metabolite regulation. Biomaterials. 2024;307:122530.

31. Prakash A, Rubin N, Staley C, Onyeaghala G, Wen YF, Shaukat A, et al. Effect of ginger supplementation on the fecal microbiome in subjects with prior colorectal adenoma. Scientific reports. 2024;14(1):2988.
32. Yang W, Yang T, Huang B, Chen Z, Liu H, Huang C. Berberine improved the microbiota in lung tissue of colon cancer and reversed the bronchial epithelial cell changes caused by cancer cells. Heliyon. 2024;10(2):e24405.
33. Huang C, Sun Y, Liao SR, Chen ZX, Lin HF, Shen WZ. Suppression of Berberine and Probiotics (in vitro and in vivo) on the Growth of Colon Cancer With Modulation of Gut Microbiota and Butyrate Production. Frontiers in microbiology. 2022;13:869931.
34. Wang M, Ma Y, Yu G, Zeng B, Yang W, Huang C, et al. Integration of microbiome, metabolomics and transcriptome for in-depth understanding of berberine attenuates AOM/DSS-induced colitis-associated colorectal cancer. Biomedicine & pharmacotherapy = Biomedecine & pharmacotherapie. 2024;179:117292.
35. Ma L, Lang Y, Xin X, Zhao W, Zhou Q, Wang J, et al. Polysaccharides extracted from hawthorn (Crataegus pinnatifida) exhibiting protective effects against DSS/AOM-induced colorectal cancer in vivo. Journal of Functional Foods. 2023;107:105618.
36. Wu C, Yang J, Ye C, Wu H, Shu W, Li R, et al. Berberine attenuates 5-fluorouracil-induced intestinal mucosal injury by modulating the gut microbiota without compromising its anti-tumor efficacy. Heliyon. 2024;10(14):e34528.
